# Partially observed epidemics in wildlife hosts: modelling an outbreak of dolphin morbillivirus in the northwestern Atlantic, June 2013–2014

**DOI:** 10.1098/rsif.2015.0676

**Published:** 2015-11-06

**Authors:** Sinead E. Morris, Jonathan L. Zelner, Deborah A. Fauquier, Teresa K. Rowles, Patricia E. Rosel, Frances Gulland, Bryan T. Grenfell

**Affiliations:** 1 Department of Ecology and Evolutionary Biology, Princeton University, Princeton, NJ, USA; 2 Robert Wood Johnson Health and Society Scholars Program, Columbia University, New York, NY, USA; 3 National Marine Fisheries Service, Marine Mammal Health and Stranding Response Program, Silver Spring, MD, USA; 4 National Marine Fisheries Service, Southeast Fisheries Science Center, Lafayette, LA, USA; 5 The Marine Mammal Centre, Sausalito, CA, USA; 6 US Marine Mammal Commission, 4340 East West Highway, Bethesda, MD, USA; 7 Fogarty International Center, National Institutes of Health, Bethesda, MD, USA

**Keywords:** dolphin morbillivirus, Poisson process, epidemiology, ecology, statistical modelling, marine mammal pathogen

## Abstract

Morbilliviruses cause major mortality in marine mammals, but the dynamics of transmission and persistence are ill understood compared to terrestrial counterparts such as measles; this is especially true for epidemics in cetaceans. However, the recent outbreak of dolphin morbillivirus in the northwestern Atlantic Ocean can provide new insights into the epidemiology and spatio-temporal spread of this pathogen. To deal with uncertainties surrounding the ecology of this system (only stranded animals were observed), we develop a statistical framework that can extract key information about the underlying transmission process given only sparse data. Our self-exciting Poisson process model suggests that individuals are infectious for at most 24 days and can transfer infection up to two latitude degrees (220 km) within this time. In addition, the effective reproduction number is generally below one, but reaches 2.6 during a period of heightened stranding numbers near Virginia Beach, Virginia, in summer 2013. Network analysis suggests local movements dominate spatial spread, with seasonal migration facilitating wider dissemination along the coast. Finally, a low virus transmission rate or high levels of pre-existing immunity can explain the lack of viral spread into the Gulf of Mexico. More generally, our approach illustrates novel methodologies for analysing very indirectly observed epidemics.

## 1. Introduction

In July 2013, an outbreak of dolphin morbillivirus (DMV) was officially declared along the northwestern (NW) Atlantic coast of the United States and has since been implicated in the stranding of over 1600 common bottlenose dolphins (*Tursiops truncatus*, hereafter referred to as bottlenose dolphin). DMV, a member of the *Morbillivirus* genus that also includes measles, rinderpest, phocine distemper virus (PDV) and canine distemper virus, infects at least 14 odontocetes species worldwide and is a major cause of morbidity and mortality [1–5]. The current outbreak has been declared an unusual mortality event (UME) due to the high number and unexpected nature of the strandings that have occurred [6]. In fact, this outbreak represents the largest number of bottlenose dolphin strandings reported in the NW Atlantic since the last recorded DMV epidemic in 1987–1988, when between 10 and 50% of the coastal population was believed to have died [7–9].

The underlying dynamics and persistence of morbilliviruses in marine mammals are poorly understood, especially for cetaceans. A variety of epidemiological dynamics have been observed in DMV hosts, from endemic transmission among pilot whales (*Globicephala* spp.) in the southwestern Pacific Ocean to recurrent epidemics among dolphins in the Mediterranean Sea, Atlantic Ocean and Gulf of Mexico (GoM) [2], but the mechanisms underlying these different patterns remain elusive. Key questions regarding the epidemiology of the virus in different cetacean species, and transmission of the virus between species, have yet to be answered. However, the current NW Atlantic UME provides a unique opportunity to explore the spatio-temporal spread of DMV within a species, and thus gain new insights into the dynamics of this poorly understood pathogen.

Mathematical and statistical modelling can help gain insight into the important drivers of disease dynamics in marine systems, but one limiting factor is the availability of detailed population and epidemiological data. For example, successful modelling of PDV outbreaks in North Sea harbour seals (*Phoca vitulina*) enabled estimation of key epidemiological parameters such as the basic reproduction number, *R*_0_ (the average number of secondary cases caused by an infected individual in a completely susceptible population) [10–13]. However, such work was aided by the ‘haul-out’ behaviour of harbour seals: individuals spend a significant amount of time aggregating on land, which enables accurate counts of population densities and individual mortalities to be made [12]. In contrast, much less is known about the coastal bottlenose dolphin populations of the NW Atlantic and no modelling work has been done that explicitly explores the drivers of DMV dynamics in this system. Population estimates are available, but some of the stocks are highly mobile and there is limited information regarding their spatio-temporal distribution and the extent of mixing within and between different stocks.

The natural history of DMV in cetaceans is also a major source of uncertainty. As with other morbilliviruses, immunity following recovery from infection is likely to be long-term [5,14], but other key epidemiological parameters such as *R*_0_, the virulence of the virus, and the duration of infectiousness are largely undocumented. Transmission is mainly thought to occur when susceptible individuals inhale aerosolized virus particles from infected individuals, possibly when close-knit groups form for travelling and feeding [15]. However, vertical transmission and direct transmission through bodily fluids may also be possible [15]. Other important quantities for DMV spread that have still to be determined include the distance an individual is likely to travel while infectious, and the spatio-temporal distribution of the susceptible population.

An additional question regarding the transmission dynamics is whether the risk of infection scales with host density (often referred to as density-dependent transmission), or remains independent of host density (frequency-dependent transmission) [16]. Density-dependent transmission may be favoured by the respiratory means of infection coupled with the gregarious behaviour of dolphins [5,15]. However, if the average number of contacts of an individual remains relatively constant in the face of host density change, then frequency-dependent transmission may be more appropriate [10,16]. Bottlenose populations inhabiting estuarine waters in the NW Atlantic are thought to maintain stable group sizes which may favour frequency-dependent transmission [17], but the degree to which this accurately captures transmission dynamics in the coastal populations is unclear.

The above uncertainties present challenges when designing an appropriate epidemic model, and so we construct a minimal stochastic framework based on a self-exciting Poisson process that requires only limited information to describe the spatio-temporal dynamics of the system. Such models are more commonly used to capture the spatio-temporal clustering of earthquakes [18] and have also been employed to model urban crime distribution [19]. However, there is great potential for application within an infectious disease context as the local nature of transmission naturally leads to clustering of cases in space and time [19–21]. We demonstrate how this method can be used to infer epidemiological parameters such as the range of host movement, the duration of infectiousness and the effective reproduction number, *R* (the average number of secondary cases caused by an infected individual in a population for which there may be pre-existing immunity). Furthermore, since the spatio-temporal pattern of strandings is likely influenced by the migrating habits of the different coastal stocks and the intensity of transmission within and between these stocks, we reconstruct transmission networks that allow us to explore the importance of host mobility in facilitating spread along the coast. Finally, we simulate an epidemic in the GoM to explore why the current UME has seemingly failed to trigger DMV-related mortality in this neighbouring region [22]. Overall, our findings provide a first step towards better understanding *Morbillivirus* spread in the NW Atlantic.

## 2. Material and methods

### 2.1. Data

Level A stranding records from 1989 to 2014 were extracted from the National Oceanic and Atmospheric Association Marine Mammal Health and Stranding Response Program (NOAA MMHSRP) National Database. For each reported stranding, the authorized responding agency is required to complete a Marine Mammal Stranding Report—Level A data form (available at www.nmfs.noaa.gov/pr/pdfs/health/levela.pdf) that includes details such as the species, date and location of each stranding. The final data used in this paper were extracted from the database on 23 May 2014 and 30 June 2014, and included strandings up through June 2014. Due to the ongoing nature of the UME at the time of writing, subsequent records may have been added that are not included in our data. Before 1996, annual observed stranding numbers were low relative to subsequent years, reflecting the absence of recorded data south of North Carolina and high variability in reporting along the remaining coastline (electronic supplementary material, figure S1). We therefore excluded these years from further analysis to minimize historical reporting bias. The subsequent stranding data (aggregated by day and latitude band as in all analyses) are available online (Github repository: http://github.com/SineadMorris/Dolphin-morbillivirus).

During the current UME, strandings have occurred along the East Coast of the United States, from New York to Florida (27–42° N latitude; electronic supplementary material, figure S3). Individuals are thought to be from the main NW Atlantic coastal bottlenose dolphin stocks: the Northern Migratory Coastal Stock (NMCS), the Southern Migratory Coastal Stock (SMCS), the South Carolina–Georgia Coastal Stock (SCGCS) and the Northern Florida Coastal Stock (NFCS), with population estimates of 11 548, 9173, 4377 and 1219 individuals, respectively [23]. However, there are other resident bay and estuarine stocks in this region, and the true origin of stranded cases has still to be confirmed by genetic analyses [23].

One caveat to these data is that the total number of infected individuals is likely to be underestimated due to inherent difficulties associated with monitoring disease outbreaks among marine mammals: generally only those animals that strand onshore can be reliably identified as infected cases and a larger proportion of animals may die at sea and not be counted in the stranding records. As such, we refer to this as a partially observed epidemic process and apply a minimal stochastic framework (outlined in §2.3) that enables the spatio-temporal dynamics of the system to be modelled despite the limited available data.

### 2.2. Data preparation

**Figure 1.**
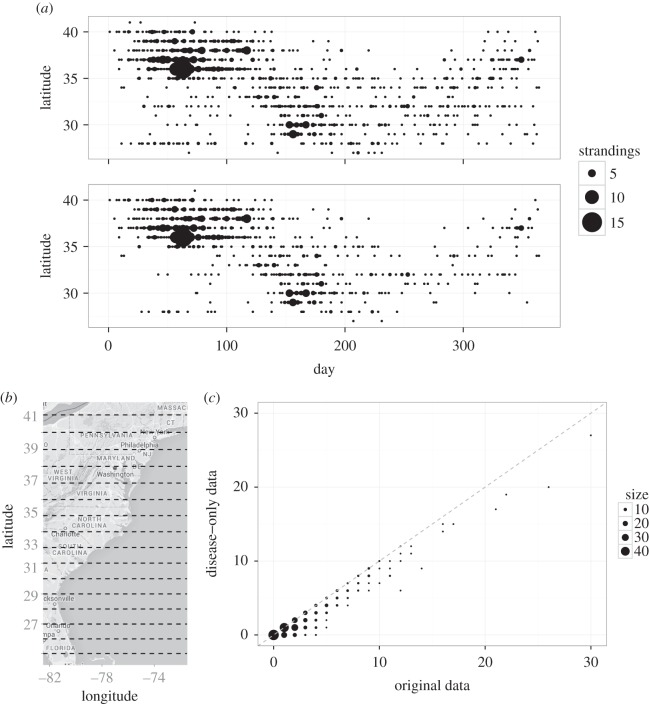
(*a*) Strandings by latitude and day since the start of the epidemic (from June 2013 to 2014) for the original UME data (top panel) and the new epidemic dataset generated by removing background cases (bottom panel). The size of each point indicates the number of strandings. (*b*) Map of the United States NW Atlantic coast with latitude bands highlighted by dashed lines (for reference). (*c*) Comparison of the new epidemic (disease-only data) and original UME datasets. Each point compares the number of strandings on a given day and latitude band between both datasets, with size indicating the number of times that particular coordinate combination occurred. The dashed line indicates when both are equal.

There was no recorded outbreak of morbillivirus infection during 1996–2012 and so these data reflect strandings that would happen in any given year, independent of DMV-related mortality. We refer to these as ‘background’ strandings and assume that the current UME data include background cases in addition to cases due to DMV. In order to implement our epidemic model, these background cases must first be removed from the UME dataset. To do this, we applied a Poisson generalized linear model to the 1996–2012 data to estimate the average annual rate of background strandings (by season and latitude degree) in non-epidemic years, and then removed the corresponding proportion of strandings from the UME dataset (see electronic supplementary material, S2.1–S2.2, for further details). This procedure preserves the overall spatio-temporal pattern of strandings when compared with the original dataset, but contains fewer sporadic cases and shows a clearer shift in the main stranding density as time progresses (figure 1*a*). We assume that potential biases in the UME data arising from non-uniform temporal and/or spatial distributions of background strandings (e.g. due to seasonal changes in the spatial distribution of host abundance) are accounted for by removing these from the data. We thus conclude that the resulting dataset sufficiently approximates the distribution of disease-induced strandings only, and it is henceforth referred to as the ‘epidemic’ dataset.

Implementation of our model also requires estimation of the spatio-temporal distribution of the NW Atlantic coastal bottlenose dolphin population. The two largest coastal stocks are believed to be migratory: the NMCS occupies waters as far north as New York (approx. 41° N) in summer and as far south as central North Carolina (approx. 34.5° N) in winter, and the SMCS ranges from Virginia (approx. 38° N) in summer to northern Florida (approx. 28° N) in winter [23–25]. However, there is large uncertainty in the precise boundaries of these ranges and how seasonal migration of these stocks affects the spatio-temporal distribution of the entire coastal population. For instance, the smaller coastal stocks (SCGCS and NFCS) are thought to be resident, i.e. non-migratory, but may come into contact with the SMCS at certain times during the year.

Previous work has made progress in this area by using stranding distributions in non-epidemic years to inform hypotheses about the structure and migration patterns of the coastal populations [25]. Guided by this approach, we assumed that the number of background strandings at any location was proportional to the local population density at the time of the strandings. This enabled us to estimate the spatio-temporal distribution of population density along the coast in a typical, non-epidemic year using the seasonal distribution of background strandings and estimates of the total coastal population size (see electronic supplementary material, S2.3, and figures S2 and S5, for further details). Our estimates are in broad agreement with the seasonal migratory ranges described above and are thus used in the epidemic model.

### 2.3. Epidemic model

We assume that the stranding locations along the coast are uniquely defined by their respective latitude coordinates, so that one spatial dimension, *l*, is sufficient to model the epidemic process. In addition, we assume that both the duration of time and the distance travelled from each individual's initial infection (unobserved data) to their eventual stranding (observed data) remain constant throughout our dataset (e.g. any pair of individuals that strand 2 days apart are assumed to have been infected 2 days apart). This enables us to approximate the spatio-temporal distribution of cases in the unobserved infection process using the distribution of strandings in our epidemic dataset.

If the full temporal and spatial ranges covered in our epidemic data are defined by 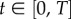 and 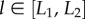, respectively, then the distribution of cases, *S_t_*_,*l*_, is modelled as an inhomogeneous (or ‘non-stationary’) Poisson process [26], for which the probability of observing *N* total cases is given by
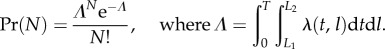


The hazard function (or conditional intensity) at time *t* and latitude *l*, *λ*(*t*, *l*), represents the rate at which new cases arise at each new point in the epidemic and is analogous to the force of infection in standard epidemiological models [19].

The history of all cases *i*, in the epidemic process up to time *t*, is defined by the set of all observation times {*t_i_*} that occur before *t* and the corresponding latitude, *l_i_*, at which the observation was recorded, i.e. *H*(*t*) = {(*t_i_*, *l_i_*)|*t_i_* < *t*} [18]. Given this history, *λ* can be expressed as
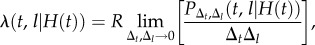
where 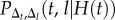 is the combined probability density that each previous case generates a new case in the time interval (*t*, *t* + Δ*_t_*) and latitude region (*l*, *l* + Δ*_l_*) (determined by the current infectiousness of each case), and *R* is the effective reproduction number (that may also vary as a function of time and latitude) [18,19,26]. Note that *R* represents the average number of new cases generated by each case over the entire epidemic, and so multiplying the probability density by *R* determines how these cases are distributed across space and time. Such frameworks, termed ‘self-exciting’ Poisson processes due to the influence of previous events on the distribution of future events [27], are often used to model processes exhibiting significant spatio-temporal clustering [19]. We use this framework to model the spatio-temporal distribution of stranding events in the NW Atlantic system within an epidemiological context, but other applications include modelling of earthquakes and urban crime [18,19].

Similar to previous formulations proposed for earthquake and epidemic modelling [18,28], we define probability distributions, *g*(*t*) and *f*(*l*), to describe how an individual's infectiousness decays in time and space, respectively. The hazard can then be expressed as2.1
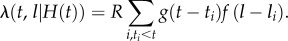


Since *t_i_* and *l_i_* are the initial observation time and location for individual *i*, respectively, it follows that (*t* − *t_i_*) is the current length of time (in days) since the beginning of that individual's infectious period and (*l* − *l_i_*) is the current distance (in latitude degrees) from the individual's initial location [18,19]. Thus, g(*t* − *t_i_*)*f*(*l* − *l_i_*) represents the current proportional contribution of the individual's overall infectiousness to the hazard at time *t* and location *l*, and the total hazard is obtained by taking the sum of the current contributions of all previously infected individuals [19,20].

Many functional forms describing the change in infectiousness are possible, but here we assume an individual's infectiousness decays exponentially in space and time from the initial infection event [20,28]. Specifically, we model the temporal decay of infectiousness using an exponential distribution,
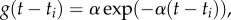
and the spatial decay with a normal distribution,
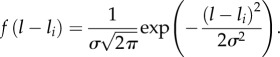


The parameters *α* and *σ* control the rate of decay of infectiousness in space and time, respectively, and are estimated during the fitting process. Although their biological interpretation is limited by the sparseness of our data, i.e. they cannot incorporate the influence of unobserved cases, they are valuable in that they allow us to characterize the dynamics of the observed infection process within an epidemiological context.

The effective reproduction number is expressed as 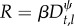, where *β* is the pathogen transmission rate, *D_t,l_* is the population size at time *t* and latitude *l*, and 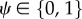 is a scaling parameter that determines whether transmission is frequency- or density-dependent. When transmission is frequency-dependent, *ψ* = 0 and *R* is constant throughout the epidemic, whereas when transmission is density-dependent, *ψ* = 1 and *R* changes in proportion to the local population distribution [16]. We investigate both scenarios in our analyses to determine which most accurately captures the underlying transmission process. We use *R* instead of *R*_0_ since we do not have sufficient evidence to assume the entire population is susceptible. Instead, we allow for the possibility that some individuals may be immune, and note that the formulation for the density-dependent model may be viewed as an upper bound for *R* as it incorporates the total population size rather than the unknown susceptible proportion.

This framework can also be adapted to capture periods where the transmission intensity may significantly fluctuate above or below the baseline value, *R*. For example, an additional reproduction number, 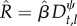, can be defined for 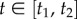 and 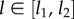 such that
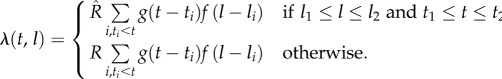


One final point is that the self-exciting Poisson framework described here does not account for the dynamic depletion of susceptible individuals over space and time as the epidemic progresses. The number of strandings on each day is small relative to the total population size and thus it is assumed that susceptible depletion will not play a significant role in the transmission dynamics. However, we also develop an analogous binomial chain model that accounts for susceptible depletion to test this assumption (see electronic supplementary material, S6, for further details).

#### 2.3.1. Parameter estimation

All parameters incorporated in the model are defined in table 1, and the log-likelihood of observing the entire time series is [19,26]
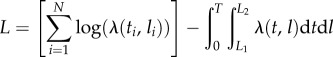

**Table 1.** Model parameters.

parameter	description
*β*	transmission rate
*D*	population density
*R*	average number of secondary infections caused by one individual
*σ*	rate of spatial decay of infectiousness
*α*	rate of temporal decay of infectiousness
*ψ*	transmission scaling factor (0 for frequency- and 1 for density-dependence)
*λ*	hazard function

(see electronic supplementary material, S3, for a derivation of this formula). To estimate the unknown parameters, *α*, *β* and *σ*, Bayesian inference was conducted via Hamiltonian Markov chain Monte Carlo (MCMC) sampling using uniform prior distributions in the R package RStan [29,30]. The effective reproduction number was calculated using estimates from the inferred parameter posterior distributions and model predictions were compared with the observed data via self-exciting Poisson simulations of the form: *S_t_*_,*l*_ = Pois(*λ_t_*_,*l*_).

Model fits were also compared using approximate Akaike information criterion (AIC) and Watanabe–Akaike information criterion (WAIC) methods, which compare the predictive accuracy of different models while penalizing those with more parameters [31–33]. Since AIC calculations rely on a point value for the maximum log-likelihood (that must be estimated from our posterior distributions), they are not entirely suited to a Bayesian framework and so we include these only for approximate comparisons. In contrast, WAIC calculations average across the posterior distribution rather than requiring a point estimate to be made and so may be more appropriate in this setting [32,33]. We thus use WAIC in conjunction with AIC to compare our different models. Further details of these information criterion calculations are given in electronic supplementary material, S4.

### 2.4. Transmission networks

Using the distribution of parameter estimates from the best-fitting model, transmission networks were reconstructed that connected stranded individuals with the source of their stranding (i.e. the previously stranded individual that most likely caused their infection). Briefly, for each *Tursiops* that stranded at time *t_i_* and latitude *l_i_*, we compared the contributions of all previously stranded individuals to the hazard at *t_i_* and *l_i_* and used the resulting probability distribution to determine the most likely source of infection (see electronic supplementary material, S7, for further details). Directed networks were then created by grouping individuals according to the latitude where they stranded. For example, a connection from node 36° N to node 38° N in the network would represent a transmission event originating from an individual that stranded at 36° N to an individual that consequently stranded at 38° N.

A different transmission network was generated for each value in a randomly sampled subset of the estimated parameter distributions, and these transmission networks then served as a tool for visualizing and interpreting the results of the model inference. In particular, network properties were evaluated using betweenness measures with the R package igraph [34]. The betweenness of a node *j* is the number of shortest paths between any pair of network nodes that pass through *j*, and thus indicates the influence or centrality of *j* within the network [35]. This measure enabled quantification of the temporal change in the spatial structure of the inferred transmission networks over the course of the epidemic.

### 2.5. Simulating an outbreak in the Gulf of Mexico

To investigate the apparent lack of viral spread to bottlenose dolphin populations in the GoM, we simulated an outbreak in this region by introducing one infected case into an otherwise susceptible population. Cases were generated according to the self-exciting Poisson process defined above, with the constraint that the cumulative number of cases could not exceed the total estimated population size. We considered only the stock that would most likely be expected to come in first contact with the currently affected NW Atlantic stocks, namely the GoM Eastern Coastal Stock that inhabits waters along the west coast of Florida from 25 to 31° N. In addition, we assumed that the total population size (7702) was equally distributed along this stretch of coastline throughout the simulation [36]. Median parameter values from the posterior distributions of the best-fitting model were used as simulation inputs.

## 3. Results

To assess whether frequency- (*ψ* = 0) or density-dependent (*ψ* = 1) transmission best characterized the underlying epidemic dynamics, both models were fitted to the data using MCMC methods as described above. There is some variation between the parameter estimates (primarily in the estimates for *R*), but the main distinction is that the frequency-dependent model returns lower AIC and WAIC values than the density-dependent model and thus represents a better fit to the entire dataset (table 2). However, the strongest epidemic signal in the data occurs during a significant peak in stranding numbers in August 2013, between southeast Virginia and northeast North Carolina (36–37° N, figure 1*a*,*b*), that is better captured by the density-dependent model (electronic supplementary material, figures S6 and S7). Thus, although frequency-dependent transmission appears to dominate in general, it is likely that there are other factors contributing to an increased transmission intensity during this peak period that the model does not account for.

**Table 2.** Model comparisons. Values indicate median parameter estimates (2.5th–97.5th quantiles) and information criterion from 2000 RStan simulations (not including warm-up sampling) of each model.

model	density-dependent	frequency-dependent	frequency-dependent with location-specific transmission rate
*R*	min: 0.045 (0.043–0.048)^a^	1.01 (0.95–1.08)	0.95 (0.89–1.02)
	max: 2.68 (2.53–2.85)		
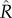	—	—	2.58 (2.08–3.17)
*σ*	1.05 (0.90–1.22)	0.78 (0.68–0.91)	0.89 (0.76–1.07)
*α*	0.11 (0.09–0.14)	0.13 (0.11–0.15)	0.12 (0.1–0.14)
number of parameters	3	3	4
approximate AIC	3821.11	3557.39	3498.09
WAIC	7358.52	7235.64	6528.21

^a^Reproductive values are calculated using the inferred posterior distribution of *β* (results not shown). The minimum value refers to *R* at the lowest population estimate for any one latitude degree and time point, and the maximum value refers to *R* at the largest population estimate.

**Figure 2.**
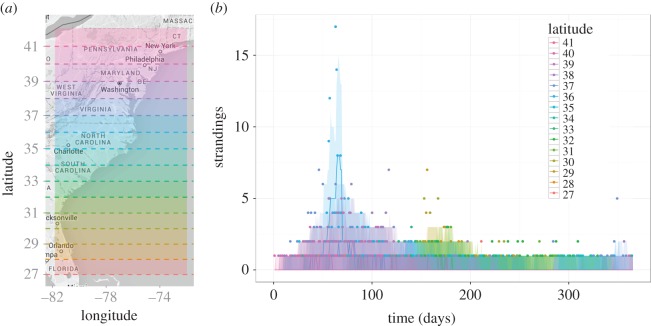
(*a*) Map of the United States NW Atlantic coast with latitude bands highlighted by dashed lines and shaded regions (for reference). (*b*) Simulated predictions of the frequency-dependent model with location-specific transmission rate. Cases were simulated across latitudes, *l*, and time, *t*, as Pois(*λ_l_*_,*t*_) using the hazard function calculated during parameter estimation. Lines represent median values from 2000 simulations in RStan, shaded regions represent the 2.5th–97.5th quantile range and points represent actual data. In both panels, latitude bands are highlighted by the same colour.

In order to better capture the key dynamics of the epidemic, we defined a new model that incorporated the frequency-dependent nature of transmission while also allowing the significant peak in August 2013 to be captured. Given the isolated nature of this signal, in space and in time, we adapted the frequency-dependent model to incorporate an additional transmission rate around the occurrence of the peak, between latitudes 36–37° N and days 50–70 (as outlined in §2.3). This modified model provided the best overall fit to the data (see AIC and WAIC values, table 2) and was also able to capture the isolated peak better than either the frequency- or density-dependent models alone (figure 2*b*). We thus conclude that this model represents the closest approximation to the underlying dynamical process and use this for all further analyses.

We also investigated frequency- and density-dependent iterations of a binomial chain model incorporating susceptible depletion, but in each case the model was a poorer fit (both in terms of the information criterion values and in capturing the significant peak) than the self-exciting models without susceptible depletion (see electronic supplementary material, S6, for details). This result is discussed further below.

**Figure 3.**
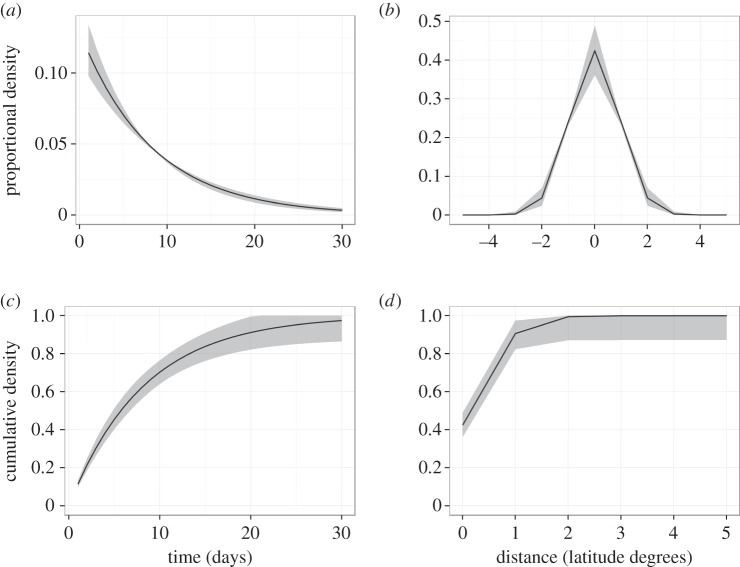
Individual infectiousness functions from the frequency-dependent model with location-specific transmission rate. Probability distributions for the temporal (*a*) and spatial decay functions (*b*). Cumulative distributions for the temporal (*c*) and spatial decay functions (*d*). Solid black lines are median values of 2000 simulations in RStan and grey shaded regions represent the 2.5th–97.5th quantile range.

The baseline estimate for the effective reproduction number, *R*, from the best-fitting model is below one (table 2), reflecting the pattern of sporadic cases that characterizes the majority of the epidemic data. The additional estimate is substantially higher (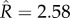), capturing an increase in transmission intensity around the main epidemic signal. The mean infectious period of an individual (1/*α*) is 8.3 days, and 95% of an individual's infectiousness is estimated to occur within 24 days and 2 latitude degrees (roughly 220 km) of the initial infection event (figure 3*a–d*). These values represent upper bounds for the likely infectious period and spatial range of an infected bottlenose dolphin. In particular, the estimated infectious period is reasonable for the generation time of a morbillivirus [10].

**Figure 4.**
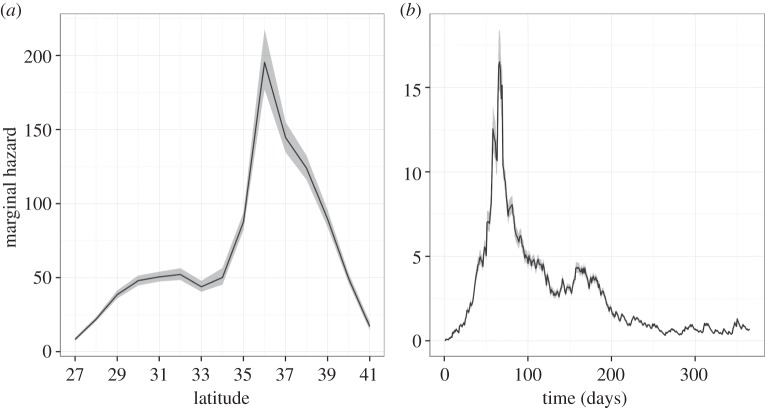
Marginal hazard distributions in space (*a*) and in time (*b*) for the frequency-dependent model with location-specific transmission rate. Solid black lines indicate median values of 2000 simulations in RStan and grey shaded regions represent the 2.5th–97.5th quantile range.

The marginal distributions for the hazard function in space and time highlight hotspots of transmission risk (figure 4*a*,*b*). Across space, there is a clear global maximum around 36–37° N, with a local plateau around 30–32° N, and across time there is one global peak towards the end of summer (at around 70 days), and another local peak near the beginning of winter (around 160 days). This suggests these particular regions and time periods are important for propagating epidemic spread and reinforces the idea that the region between southeast Virginia and northeast North Carolina may be driving an increase in transmission intensity towards the end of summer.

### 3.1. Transmission networks

Transmission networks were generated as outlined in §2.4. Transmission events resulting from movements of the order of 1–2 latitude degrees are most common (in accordance with our spatial range estimates), although long-range movements do occur and are important for seeding events in regions where there are no (or relatively few) infections (e.g. electronic supplementary material, figure S10). Therefore, high mobility of some individuals (e.g. during migration) may explain the wide dissemination of the virus along the coast.

**Figure 5.**
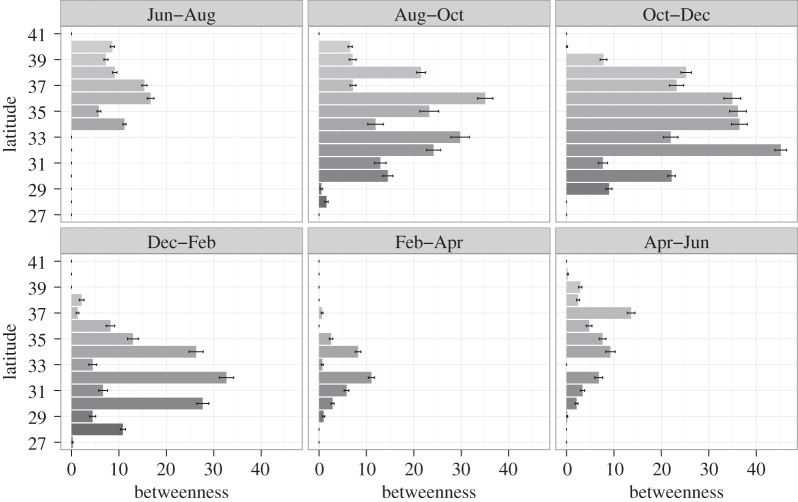
Betweenness of each latitude band in the reconstructed transmission networks across bimonthly intervals, starting 26 June 2013 (darker colours represent more southerly latitude points). Betweenness measures the number of shortest paths running through a particular node for each of the sampled networks. Mean values (grey shaded bars) and standard error bars (black lines) are calculated from 100 sampled network trees.

To explore this further, we measured temporal changes in the betweenness of the network as an indication of the relative importance of each latitude degree in propagating the underlying transmission process. In general, the most influential nodes at any time point are clustered around the regions where transmission is greatest (figure 5). As would be expected, the nodes with the highest initial betweenness are those clustered around the northern latitudes where the epidemic is believed to have begun, in particular the 36–37° N region. Following the most influential nodes throughout the year then signals how the centre of mass of the epidemic changes: the highest betweenness values gradually shift to the more southerly regions during winter and autumn, before moving northwards again during the warmer months. This again suggests that seasonal migration is likely to play a key role in the spatial spread of the epidemic.

### 3.2. Gulf of Mexico

**Figure 6.**
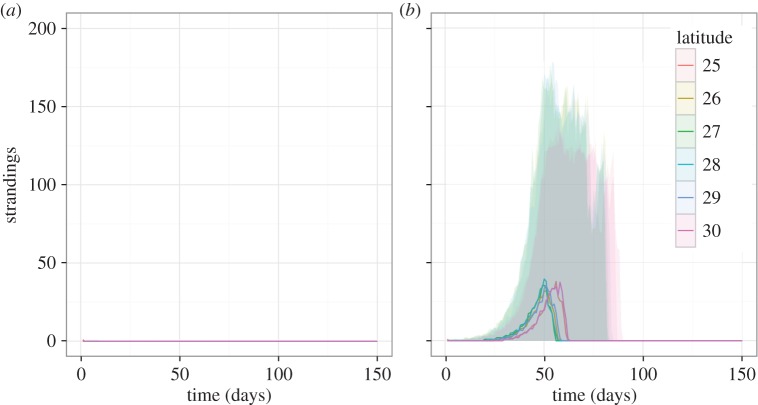
Simulations of an outbreak initiated in the GoM using parameters obtained from the best-fitting model. (*a*) Best-case scenario using the baseline reproduction number (*R*_0_ = 0.95). (*b*) Worst-case scenario using the increased reproduction number (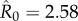). Solid lines represent median values of 100 simulations and shaded regions represent the 2.5th–97.5th quantile range. Latitude bands are distinguished by colour.

We simulated an epidemic in the GoM assuming frequency-dependence and using parameter estimates obtained from the best-fitting model (as described in §2.5) to explore the likelihood of an outbreak in this region. We additionally assumed the population was entirely susceptible and so our NW Atlantic effective reproduction number estimates, *R* = 0.95 and 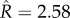, can be viewed as lower bounds for *R*_0_ and 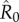 in the GoM, representing the ‘best’ and ‘worst’ case scenarios, respectively. In the best-case scenario, the outbreak always fails to take off (figure 6*a*), whereas in the worst-case scenario, although not all outbreaks take off, those that do result in rapid viral spread until all susceptibles have been infected (figure 6*b*). Although all epidemics are initiated in the lowest latitude band (where dolphins from the GoM and NW Atlantic are most likely to come in contact), peaks tend to occur first in the intermediate latitudes as a result of the uniform distribution of individuals and the additional force of infection gained from being surrounded by infected neighbours.

To investigate potential levels of pre-existing immunity in the GoM, we use standard epidemiological theory which states that *R* must be less than one to avoid an outbreak (where *R* = *R*_0_ × *s* and *s* is the proportion of the population that are susceptible) [16]. In the best-case scenario, *R*_0_ < 1 and so an outbreak will never take off. In the worst-case scenario, 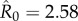 and so
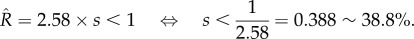


This suggests at least 61.2% of the population should be immune to avoid an outbreak.

## 4. Discussion

The aim of this paper was to gain insight into the spatio-temporal spread of DMV in the NW Atlantic using data that may represent only partially recovered *Tursiops* mortality. With a self-exciting Poisson framework, we were able to estimate previously unknown epidemiological parameters and reconstruct networks to visualize key aspects of the underlying transmission process. This has provided a greater understanding of the ecology of disease spread among the dolphin stocks in this region.

### 4.1. Model fits and parameter estimates

The best-fitting model assumed frequency-dependent transmission, which agrees with previous findings for PDV and measles [10,11,37]. Frequency-dependence suggests that transmission is largely independent of population density, and typically results from populations with stable social structures in which individuals generally have a fixed number of contacts (where ‘contact’ refers to interactions that may lead to disease transmission) [10,16]. However, at this stage, we cannot rule out density-dependent transmission fully, as the relatively poor overall fit of that model could be due to local inaccuracies in our population distribution estimates (which rely on historical stranding data and the assumption that stranding numbers in a particular area are proportional to the local population density). In particular, the density-dependent model captured the outlying peak in cases better than the original frequency-dependent model (with constant transmission intensity). This is most likely due to the large population sizes predicted in this region that increase the local hazard of the density-dependent model and thus allow more cases to be generated. More detailed data on stock movement, social structures and virus transmission pathways are thus needed to further understand the scaling of transmission in this system.

The estimate for the baseline effective reproduction number (*R* = 0.95) suggests low-intensity transmission and is consistent with the occurrence of sporadic cases and the absence of a characteristic epidemic growth phase. Allowing an additional transmission rate around the outlying peak (36–37° N, August 2013) increased the model fit and resulted in a substantial increase in transmission intensity (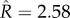). One driver of this increase may be seasonal migration. Around Virginia Beach, VA (a section of coastline within this 36–37° N region), Barco *et al.* [38] found substantially higher numbers of *Tursiops* in August than at any other time of year, potentially due to the overlapping of the NMCS and SMCS at the extreme boundaries of their migratory ranges. This aggregation of individuals at higher densities could cause nonlinear increases in the frequency of contact between hosts that would explain the subsequent jumps in transmission intensity that were not captured by the model with constant transmission parameter. Moreover, our additional estimate falls within the range of reproduction number estimates for PDV (2–3), for which high aggregation of seals at haul-out sites was believed to facilitate rapid virus transmission [11].

The infectious period of an individual had an upper bound of 24 days, with a mean of 8 days. This is similar to other morbillivirus infections, including measles (6–9 day latent period and 6–7 day infectious period) and PDV (11–18 day combined latent and infectious period) [10,11,39]. Two latitude degrees (approx. 220 km) was the upper bound for the expected travel distance of an individual during their infectious period; further distances were possible, but rare. The coastal stocks exhibit both migratory and sedentary behaviour (depending on the time of year) and thus movement patterns are highly variable and difficult to quantify. However, our estimated ranges are supported by movement data from tagged NMCS individuals that show weekly travel distances of up to 347 km (mean: 114 km; 2.5th–97.5th quantiles: 2–299 km; *N* = 240) (A. Hohn 1998–2000, unpublished data). We note that the spatial and temporal decay parameters (*σ* and *α*) may be overestimated as our model does not account for the influence of unobserved cases. However, it is encouraging that our estimates do not vary substantially between the different models tested and that they are within such close ranges of the corresponding quantities discussed above from related viruses (measles and PDV) and the population movement data.

The inference process was also applied to frequency- and density-dependent models incorporating susceptible depletion, but the overall fits to the data were worse than both the frequency- and density-dependent Poisson processes. There are two likely reasons for this. First, since only reported strandings are included in the analysis and the total number of these observed cases is small relative to the total size of the NW Atlantic population, new infections are unlikely to significantly alter the available pool of susceptible individuals in our models. Second, our models do not explicitly account for the extent and distribution of pre-existing immunity to DMV, or the role of host migration in shaping transmission dynamics and the subsequent spatio-temporal distribution of susceptibility. These factors should strongly influence the importance of susceptible depletion in this system, but due to lack of information could not be included in the current framework without compromising model parsimony. A greater understanding of the underlying spatio-temporal distribution of susceptibility to DMV would enable future modelling to incorporate susceptible depletion in a more realistic framework.

### 4.2. Transmission networks

Analysis of the reconstructed transmission networks indicated that certain regions may disproportionately promote virus transmission at different times of the year, and long-range movements of dolphins are important for seeding the epidemic in new, unaffected regions. Measuring betweenness at discrete intervals suggested a seasonal shift in the centre of gravity of the underlying transmission process, further supporting the idea that stock migration is a key factor in propagating virus spread along the coast [24]. In particular, Rosel *et al.* [24] proposed that the seasonal spread of the 1987–1988 epidemic was a result of the virus being transferred from the NMCS to the SMCS following interaction between the two stocks at times of population overlap. More detailed information on the seasonal movements of the coastal stocks is needed to investigate this further.

### 4.3. Gulf of Mexico

Assuming the virus was introduced to bottlenose dolphins in the GoM (e.g. through contact with the NW Atlantic stocks) and the transmission intensity was equal to our increased estimate, 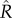, we found that an outbreak should occur unless there were high levels of pre-existing immunity in the population. The last confirmed DMV outbreak in the GoM occurred in 1994, with suspected outbreaks also occurring in 1990 and 1992 [1,40]. Given that bottlenose dolphins in this region can live up to 40–50 years, there may be survivors in the current population that have maintained immunity from these previous outbreaks [41,42]. Although Rowles *et al.* [41] found seropositivity to DMV ranging from 0 to 18% between sampling regions in the GoM, the extent and spatial distribution of evidence of previous exposure across the wider population remains unclear, and further information is needed to compare this with our 61% estimate derived for the Eastern Coastal Stock.

On the other hand, we found that an outbreak would fail to take off at the baseline transmission rate, even in an entirely susceptible population. This could also sufficiently explain the lack of substantial DMV spread since only sporadic cases would be expected in this scenario. Given the association between 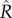 and large seasonal aggregations of individuals in the NW Atlantic, it is possible that the smaller size of the GoM Eastern Coastal Stock is insufficient to generate the frequency of contacts needed to support a transmission intensity of comparable levels. Further modelling work could help to identify a threshold population size above which density-dependent effects may become important in this region.

Seasonal migration and contact between different stocks are also key factors in promoting viral dissemination along the coast [1]. Therefore, another reason for limited spread could be that there is simply not enough contact between the GoM and NW Atlantic stocks to provide sufficient transmission opportunities. Rosel *et al.* [24] found substantial genetic differences between NW Atlantic and GoM bottlenose dolphins that was consistent with limited mixing between the two populations. However, given that an infected individual stranded as far south as the Florida Keys in November 2014 [43], there is clear potential for viral spread into the neighbouring GoM. More information on the levels of contact between these two regions would enable this to be explored further.

### 4.4. Caveats and future directions

Marine mammals, and especially cetaceans, represent challenging study subjects as it is common to have only limited data regarding population sizes, individual dispersal patterns and disease prevalence [44]. We used historical stranding distributions to infer the spatio-temporal distribution of population density and assumed that the observed epidemic strandings could be used as a proxy for the underlying infection process. However, there are a number of caveats to these assumptions. Firstly, the stranding data do not include those individuals that recover from infection or those that die from the disease but do not strand onshore, and so we have only a partial representation of the infection process. The self-exciting Poisson framework provides a means of identifying key epidemiological parameters from the outbreak despite these sparse data; however, there may be important information embedded in the unobserved infection process that is not accounted for. Unfortunately, integrating an additional unobserved process into the model is challenged by identifiability issues arising from the sparseness of the data and the computational intensity of performing inference on a model with more unknown variables.

Secondly, wind, oceanic currents and the length of coastline in a particular area can affect the likelihood of an individual stranding onshore, and the location and timing of the stranding should it occur [25,45]. Using daily wind force data, Rijks *et al.* [45] found that wind had a confounding effect on the distribution of seal strandings during the 2002 PDV outbreak in the Netherlands. Similar data from the NW Atlantic could be used to assess the influence of wind and other weather variables on stranding distributions. Future work could account for the length of available coastline when calculating the distribution of population density from historical strandings and could also integrate weather components into the model as an additional term affecting the location and timing of strandings. This would enable a more realistic representation of the stranding process and could also provide greater insight into the unobserved infection process.

Thirdly, temporal and spatial heterogeneity in reporting effort can influence the likelihood and promptness of carcass identification [25]. Although this may be a significant factor in the historical stranding data, we expect reporting to be uniformly high across all locations during the current UME due to the public attention and rapid response of NOAA. Again, more detailed information on the distribution of the stocks would better inform our population estimates and decrease the likelihood of historical reporting bias influencing our findings.

In addition, we assume the population is closed and do not include the reintroduction of DMV from other marine mammal populations that have been implicated as reservoir hosts, such as pilot whales and offshore bottlenose dolphins [1,46]. Such spillover could be incorporated into the model via an additional hazard function term representing some baseline risk of external infection [20,21]. However, further information on the prevalence of DMV in other Atlantic cetaceans, and the frequency of contact between these populations and the coastal bottlenose stocks, would be needed to assess the likelihood of these events and guide the parametrization and functional form of such a term.

Our hazard function also assumes an exponential form for the spatial and temporal decay of infectiousness and does not explicitly account for individual migration. There have been more complex models of dolphin movement but these have been based on explicit tracking of individuals [47,48]. Since our stranding data contain no information on how animals move during the epidemic, we have adopted the most parsimonious framework to describe dispersal that can provide biologically interpretable results in broad agreement with existing knowledge and data. More information on how virus infectivity changes over time within a given individual and on the complex movements, migration patterns, and interactions between the different stocks in the NW Atlantic is needed to guide alternative hazard functions. In particular, tagging individuals during disease outbreaks is an important area for future work.

Finally, age-structured models have provided key insights into the influence of social structure on the spread of other morbilliviruses such as PDV and measles [11,49]. In particular, subadult harbour seals were disproportionately important in transmitting PDV during the 2002 Dutch outbreak [11]. Juvenile bottlenose dolphins tend to have a larger number of contacts than individuals in any other age class [50], and thus may similarly be important vectors driving the spread of DMV in the NW Atlantic. In addition, there may be differences in stranding times between age classes that could only be accounted for in an age-structured model. Estimates of age for stranded *Tursiops* (measured by length) are available from the current UME dataset. This would therefore be another interesting covariate for future analyses.

In conclusion, despite many unknowns surrounding the spread of DMV in cetaceans, we have gained important insights into the epidemiology of this pathogen by using a simple statistical framework to model the current NW Atlantic UME. Furthermore, there is clear potential to extend this methodology beyond the current framework (e.g. [20,21,28,51–54]), although requirements for future applications will depend on the system under consideration, in particular on the resolution and spatio-temporal patterns of the observed data, and the complexity of the desired hazard function for capturing underlying disease dynamics. Overall, however, we believe this work illustrates the general utility of self-exciting Poisson models to analyse partially observed spatial epidemics. Future serological studies, migration data and population census information would enable more rigorous testing of the key findings outlined in this work and provide a platform for the development of more sophisticated frameworks to model the underlying system dynamics.

## Supplementary Material

Data (section 1). Methods (sections 2 - 4). Results (sections 5 - 7)
