# Supplementary material for: Partially observed epidemics in wildlife hosts: modelling an outbreak of dolphin morbillivirus in the northwestern Atlantic, June 2013–2014
Source: J R Soc Interface. 2015 Nov 6;12(112):20150676. doi: 10.1098/rsif.2015.0676 (PMC4685842; doi:10.1098/rsif.2015.0676)
Supplement: Data (section 1). Methods (sections 2 - 4). Results (sections 5 - 7) [file rsif20150676supp1.pdf]

PARTIALLY OBSERVED EPIDEMICS IN WILDLIFE HOSTS: MODELING AN OUTBREAK OF DOLPHIN MORBILLIVIRUS IN THE NORTHWESTERN ATLANTIC, JUNE 2013–2014

Sinead E. Morris[1*†], Jonathan L. Zelner[2*], Deborah A. Fauquier[3], Teresa K. Rowles[3], Patricia E. Rosel[4], Frances Gulland[5,6], Bryan T. Grenfell[1,7].

[1] Department of Ecology and Evolutionary Biology, Princeton University, Princeton, NJ, USA.
[2] Robert Wood Johnson Health and Society Scholars Program, Columbia University, New York, NY, USA.
[3] National Marine Fisheries Service, Mammal Health and Stranding Response Program, Silver Spring, MD, USA
[4] National Marine Fisheries Service, Southeast Fisheries Science Center, Lafayette, LA, USA
[5] The Marine Mammal Centre, Sausalito, CA, USA
[6] U.S. Marine Mammal Commission, 4340 East West Highway, Bethesda, MD, USA
[7] Fogarty International Center, National Institutes of Health, Bethesda, MD, USA.
[*] Joint first author
[†] Corresponding author: S. E. Morris; semorris@princeton.edu

Supplementary Information

S1. Stranding data 2

S2. Data preparation 3

S2.1. Prediction of background stranding rates 3

S2.2. Removing background strandings from the UME dataset 4

S2.3. Seasonal distribution of population density 4

S3. Log-likelihood function 7

S4. Information criterion 8

S5. Frequency vs. density-dependent model predictions 8

S6. Binomial chain model with susceptible depletion 9

S7. Transmission networks 11

S7.1. Generating the networks 11

S7.2. Visualization 12

References 13

S1. Stranding data

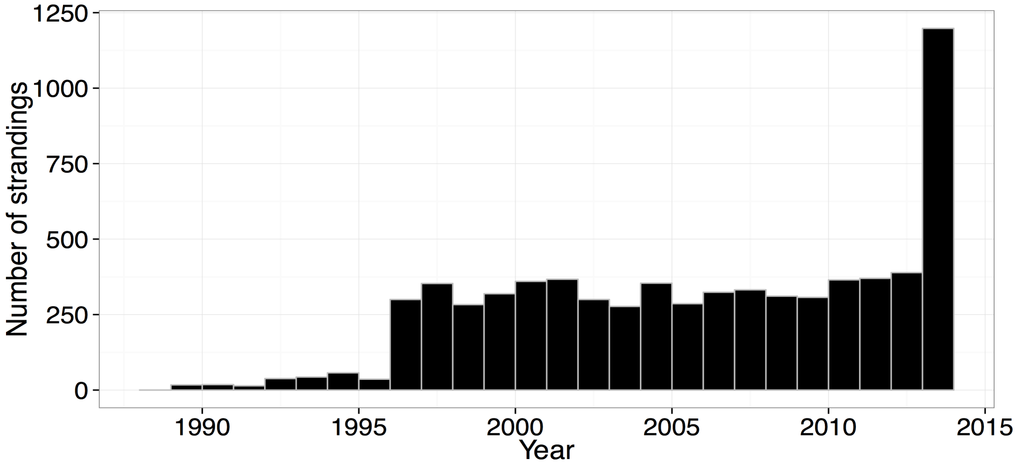

Figure S1. Number of strandings by year between 27–42°N. Data were obtained from the MMHSRP National Database (accessed 23 May and 30 June 2014). Low numbers prior to 1996 suggest low reporting rates and so are discarded in all subsequent analysis. Similarly the spike in strandings in 2013 reflects the beginning of the UME, and so we do not include this year when considering 'non-epidemic' years.

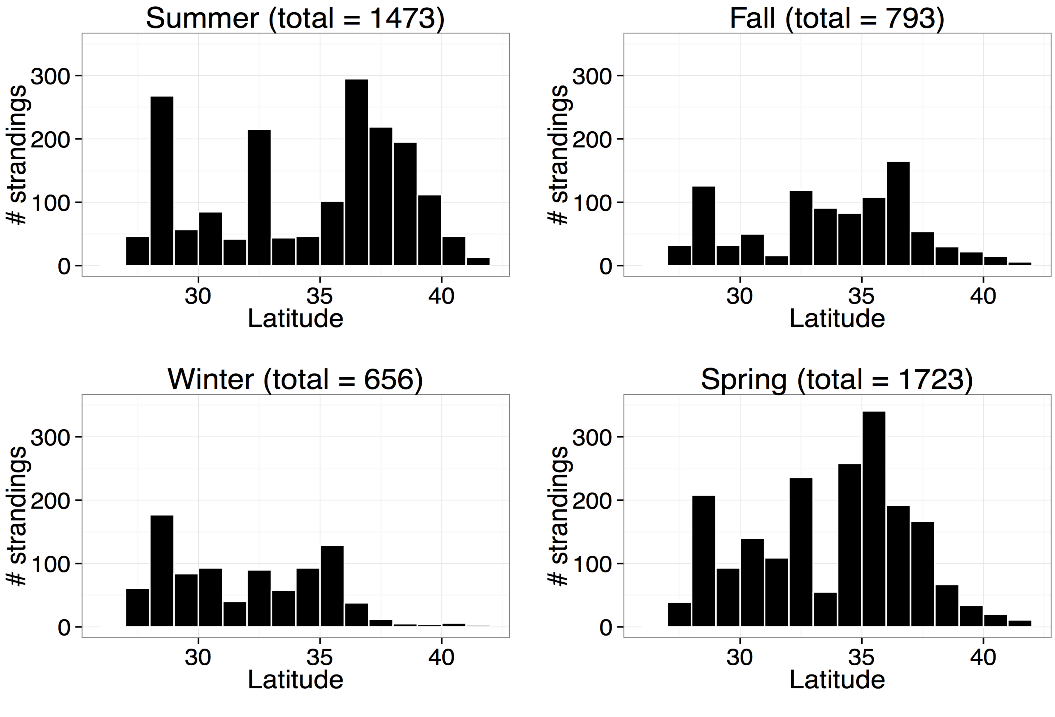

Figure S2. Number of strandings by season and latitude degree from 1996–2012. Data were obtained from the MMHSRP National Database (accessed 23 May and 30 June 2014). September, October and November are classed as fall months; December, January and February as winter; March, April and May as spring; and June, July and August as summer.

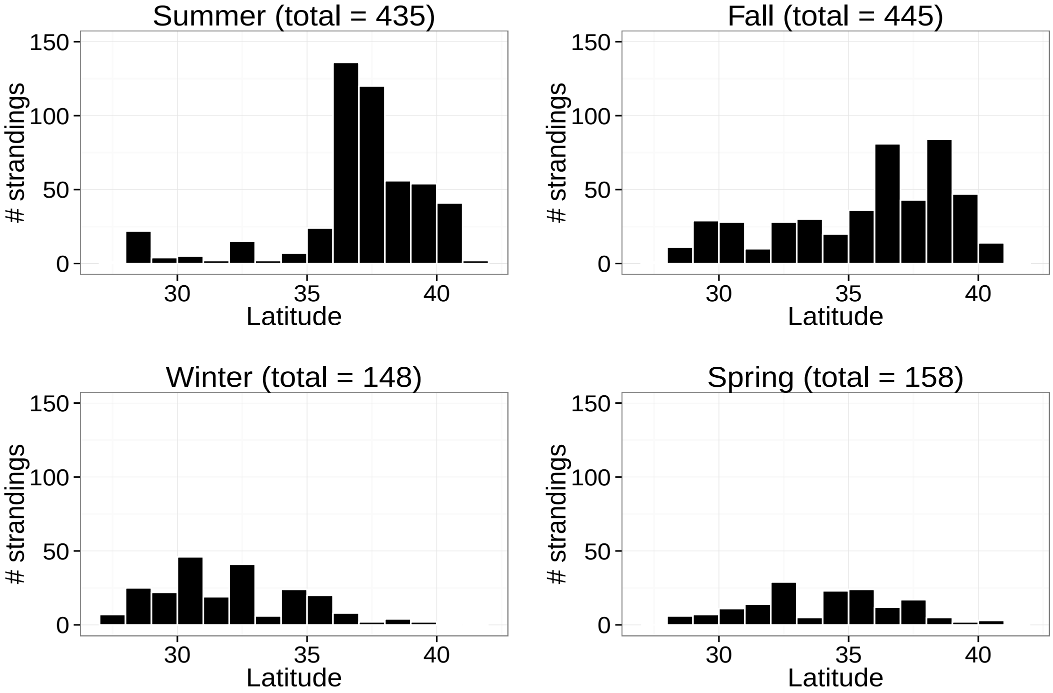

Figure S3. Number of strandings by season and latitude degree for the UME period (June 2013–2014). Data were obtained from the MMHSRP National Database (accessed 23 May and 30 June 2014). September, October and November are classed as fall months; December, January and February as winter; March, April and May as spring; and June, July and August as summer.

S2. Data preparation

S2.1. **Prediction of background stranding rates.** Annual stranding rates in non-epidemic years (1996–2012) were predicted using a Poisson generalized linear model (GLM). Separate models for summer (June–August), fall (September–November), winter (December–February) and spring (March–May) were used to allow for seasonal variability in stranding patterns (Figure S2). Each model was defined by

$$\theta_s = \exp{(b_0 + b_1 L)}, \qquad N_s \sim \text{Pois}(r_s)$$

where L is a vector representing latitude degrees, N_s a vector for the total number of strandings at each latitude degree in a given season, s, and r_s a vector for the stranding rate at each latitude during season s.

The resulting predictions for the number of background strandings in each season and latitude degree (in the absence of a disease outbreak) sufficiently capture the observed seasonal patterns in the background stranding data, with spring and summer displaying higher stranding rates in addition to a stronger multimodal pattern in the distribution of rates across latitude points compared to fall and winter (Figure S4 and Table S1). We therefore use these estimates to separate likely background cases in the 2013 – 2014 UME data from cases that are likely due to disease (Section S2.2), and to estimate the seasonal distribution of the total population along the coast (Section S2.3).

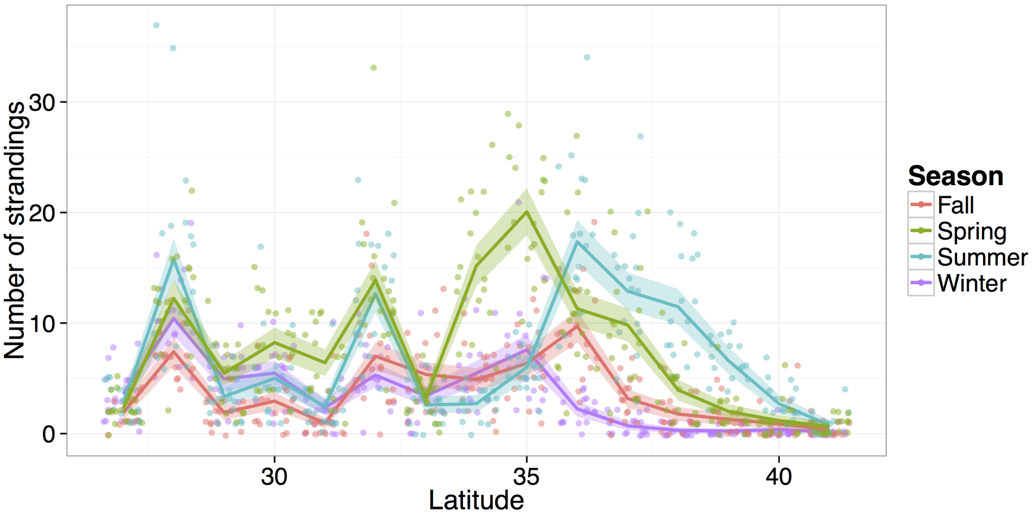

Figure S4. Poisson GLM fit of background strandings by season and latitude for non-epidemic years (1996–2012). Points represent observed number of strandings within each latitude band, for each year and season; coloured lines represent predicted annual stranding rates for each season and latitude band; and shaded regions are the corresponding 95% confidence intervals.

S2.2. **Removing background strandings from the UME dataset.** As discussed in Section 2.2, the background cases must be removed from the UME dataset in order to restrict analysis to strandings due to DMV infection. Since we only consider the first year of UME data in our analysis, the number of recorded UME strandings in each season and latitude is analogous to the annual UME stranding rate. These UME stranding rates, in addition to the background stranding rates calculated previously, are used to determine the proportion of cases that should be removed from the UME dataset.

Let $r_{s,l}$ and $u_{s,l}$ be the rate of background and UME strandings in season s and latitude l, respectively. Note that since the UME data includes background strandings, $r_{s,l} < u_{s,l}$. Assuming the stranding events are independent, the probability that a randomly selected case from season s and latitude l in the UME dataset is a background stranding follows a binomial distribution with 'success' probability $r_{s,l}/u_{s,l}$. The successful outcomes are then removed from the UME dataset. Figure 1A compares the raw dataset to the resulting epidemic dataset once these background cases have been removed. The pattern shown here is preserved across multiple simulations of the above procedure and we therefore assume that our approximation of the epidemic data is a fair representation of the underlying epidemic process.

S2.3. **Seasonal distribution of population density.** If N_s is the the total number of strandings (across all latitude degrees) during a particular season s, and $N_{s,l}$ is the number of strandings at latitude l during that given season, then the proportion of strandings in season s that occur at latitude l is $N_{s,l}/N_s$. The number of individuals that occupy latitude l during season s can then be estimated by multiplying $N_{s,l}/N_s$ by the total population size (26,317) of the four coastal stocks (NMCS, SMCS, SCGCS and NFCS). Since it is assumed that estuarine dolphins do not contribute significantly to the overall UME strandings, their stock sizes are not included in the total population estimates or any subsequent analysis.

Table S1. Summary of Poisson GLM predictions for annual background stranding rates in non-epidemic years (1996–2012). Coefficient estimates (and standard errors) are given for each season and latitude.

	Strandings:			
	Summer	Fall	Winter	Spring
Latitude 28	1.762^{***}	1.371^{***}	1.065^{***}	1.674^{***}
	(0.160)	(0.198)	(0.148)	(0.174)
Latitude 29	0.214	−0.000	0.320^{*}	0.869^{***}
	(0.198)	(0.250)	(0.168)	(0.191)
Latitude 30	0.614^{***}	0.446^{**}	0.422^{**}	1.278^{***}
	(0.183)	(0.226)	(0.165)	(0.181)
Latitude 31	−0.091	-0.693^{**}	-0.422^{**}	1.028^{***}
	(0.213)	(0.306)	(0.203)	(0.187)
Latitude 32	1.542^{***}	1.313^{***}	0.389^{**}	1.800^{***}
	(0.162)	(0.199)	(0.166)	(0.173)
Latitude 33	−0.044	1.045^{***}	−0.050	0.344
	(0.211)	(0.206)	(0.183)	(0.209)
Latitude 34	−0.000	0.953^{***}	0.422^{**}	1.889^{***}
	(0.209)	(0.208)	(0.165)	(0.172)
Latitude 35	0.796^{***}	1.216^{***}	0.749^{***}	2.168^{***}
	(0.178)	(0.201)	(0.155)	(0.169)
Latitude 36	1.858^{***}	1.640^{***}	-0.473^{**}	1.594^{***}
	(0.159)	(0.193)	(0.207)	(0.176)
Latitude 37	1.560^{***}	0.523^{**}	-1.626^{***}	1.454^{***}
	(0.162)	(0.223)	(0.316)	(0.178)
Latitude 38	1.444^{***}	−0.065	-2.501^{***}	0.541^{***}
	(0.164)	(0.254)	(0.465)	(0.201)
Latitude 39	0.890^{***}	−0.375	-2.725^{***}	−0.137
	(0.175)	(0.277)	(0.516)	(0.235)
Latitude 40	−0.000	-0.758^{**}	-2.319^{***}	-0.668^{**}
	(0.209)	(0.313)	(0.428)	(0.275)
Latitude 41	-1.264^{***}	-1.674^{***}	-3.012^{***}	-1.266^{***}
	(0.314)	(0.445)	(0.591)	(0.341)
Constant	0.995^{***}	0.633^{***}	1.278^{***}	0.830^{***}
	(0.147)	(0.177)	(0.128)	(0.160)
Note:				$^{*}p<0.1$; $^{**}p<0.05$; $^{***}p<0.01$

The method described above gives a point estimate for the number of individuals at each latitude that remains constant throughout a given season. In reality, however, the number of individuals is likely to fluctuate continuously throughout the season. Since we lack data on these fluctuations, we incorporate them by linearly interpolating around our point estimates. For example, if D_l^F, D_l^W are the point estimates for the population size at latitude l in fall and winter, respectively, then for each day $(t = 1, 2, ..., 91)$ of fall, the number of individuals at l is given by

$$D_{t,l} = D_l^F + \frac{(t-1)}{91}(D_l^W - D_l^F).$$

This procedure is repeated for each season to give a smoother temporal function for the population density at each latitude degree (Figure S5). These estimates then provide the model inputs for the population size at each day and latitude degree, $D_{t,l}$, throughout the epidemic.

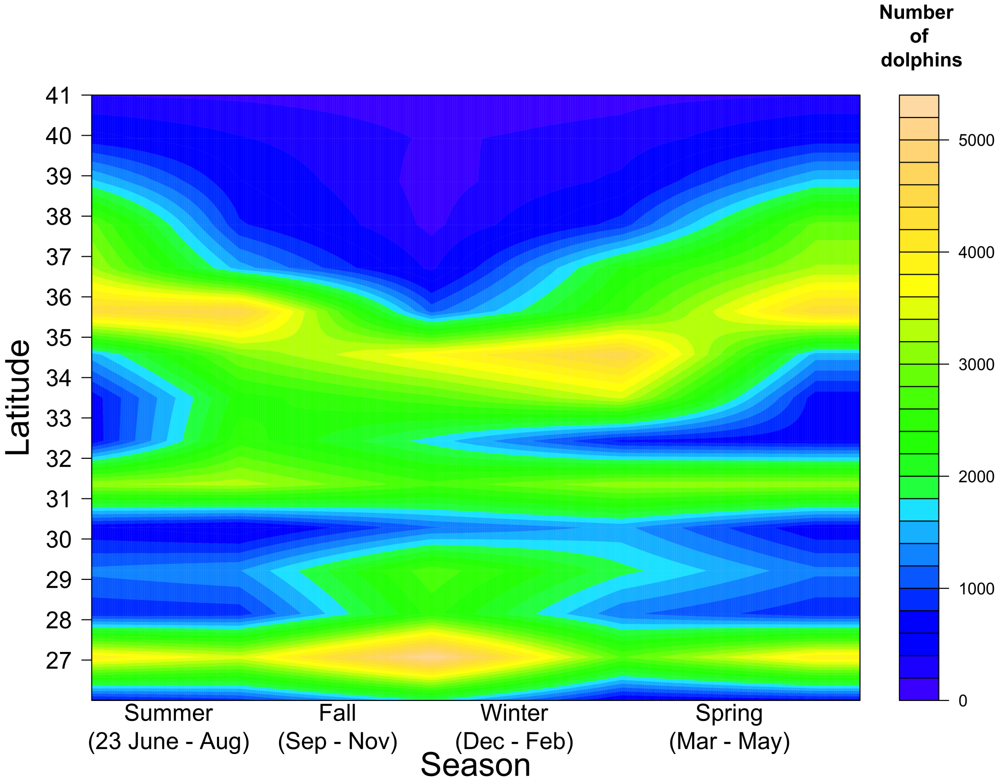

Figure S5. Estimate of the spatiotemporal distribution of coastal bottlenose dolphins in the NW Atlantic. The total population size is assumed to be 26,317, comprising the NMCS, SMCS, SCGCS and NFCS. Estimates are obtained by assuming the annual stranding rates at any latitude in non-epidemic years is proportional to the total number of individuals at that latitude at any given time (see Sections 2.2 and S2.3 for further details).

S3. Log-likelihood function

As presented in Section 2.3.1, the probability of observing N cases in time $t \in [0,T]$ and space $l \in [L_1, L_2]$ is defined as [1]

$$\Pr(N) = \frac{\Lambda^N \mathrm{e}^{-\Lambda}}{N!}, \qquad \text{where} \qquad \Lambda = \int_0^T \int_{L_1}^{L_2} \lambda(t,l)\mathrm{dtdl}.$$

The probability of observing case i is $\lambda(t_i, l_i)/\Lambda$ [1], and the probability of observing an ordered sample of cases $1, 2, ..., N$ is

$$\begin{aligned}\Pr(\text{sample}) &= N! \prod_{i=1}^{N} \frac{\lambda(t_i, l_i)}{\Lambda} \\ &= \frac{N!}{\Lambda^N} \prod_{i=1}^{N} \lambda(t_i, l_i).\end{aligned}$$

It follows that the likelihood of observing the whole epidemic dataset is given by [1]

$$\begin{aligned}\Pr(N) \times \Pr(\text{sample}) &= \frac{\Lambda^N \mathrm{e}^{-\Lambda}}{N!} \times \frac{N!}{\Lambda^N} \prod_{i=1}^{N} \lambda(t_i, l_i) \\ &= \ \mathrm{e}^{-\Lambda} \times \prod_{i=1}^{N} \lambda(t_i, l_i),\end{aligned}$$

and therefore the log-likelihood is [1, 2]

$$\begin{aligned}L &= -\Lambda + \sum_{i=1}^{N} \log(\lambda(t_i, l_i)) \\ &= -\int_0^T \int_{L_1}^{L_2} \lambda(t,l)\mathrm{dtdl} + \sum_{i=1}^{N} \log(\lambda(t_i, l_i)).\end{aligned}$$

S4. Information criterion

Typical Akaike information criterion (AIC) values are calculated as

$$\mathrm{AIC} = 2k - 2\log(p(y|\hat{\theta})),$$

where y represents the observed data, k is the number of estimated parameters in the model, and $\hat{\theta}$ is the maximum likelihood estimate of the model parameters θ [3, 4]. Here, the maximum log-likelihood value, $\log(p(y|\hat{\theta}))$, is approximated by taking the median value of the posterior log-likelihood distribution.

Watanabe-Akaike information criterion (WAIC) values are calculated as

$$\mathrm{WAIC} = 2\sum_{i=1}^{n} \mathrm{var}_P\Big(\log(p(y_i|\theta))\Big) - 2\sum_{i=1}^{n}\log\Big(\int p(y_i|\theta)p_P(\theta)\mathrm{d}\theta\Big),$$

where $p_P(\theta)$ represents the posterior parameter distribution [4, 5]. The first term sums the posterior variance of the log-likelihood for each observed data point, y_i, and approximates the number of fitted parameters, and the second term provides a measure of the pointwise log-likelihood [4].

S5. Frequency vs. density-dependent model predictions

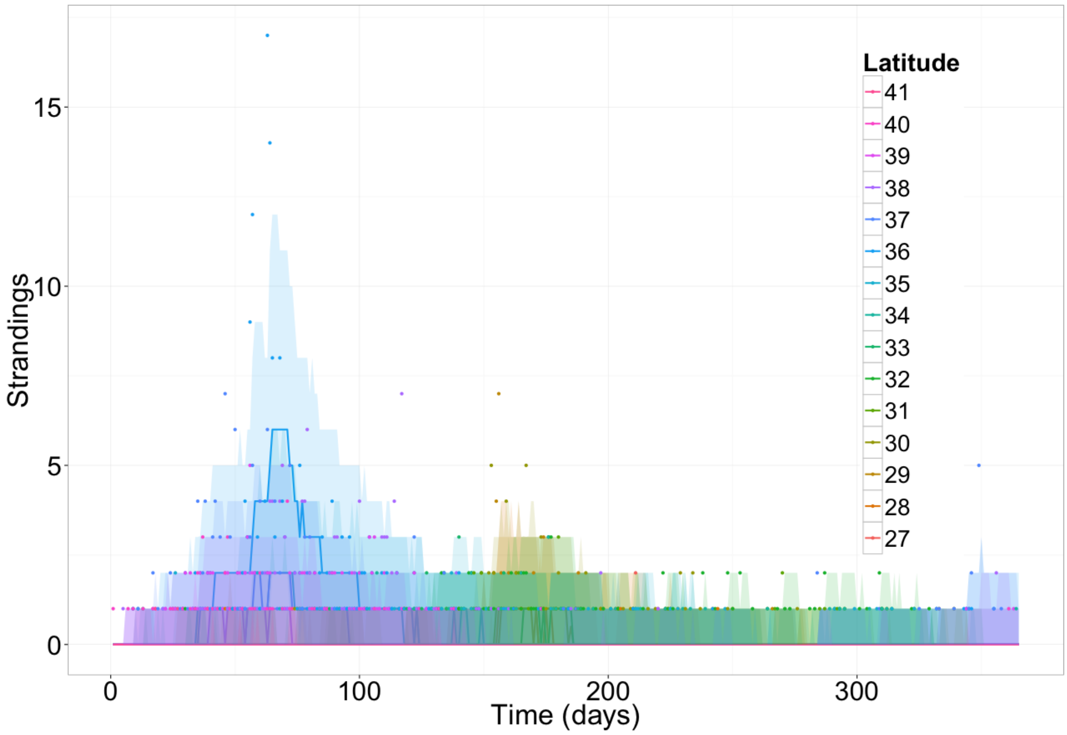

Figure S6. Simulated predictions of the density-dependent model. Cases were simulated across latitudes, l, and time, t, as $\mathrm{Pois}(\lambda_{l,t})$ using the hazard function, $\lambda_{l,t}$, calculated during parameter estimation. Lines represent median values from 2000 simulations in RStan, shaded regions represent the 2.5^{th}–97.5^{th} quantile range and points represent actual data. Latitude bands are distinguished by colour.

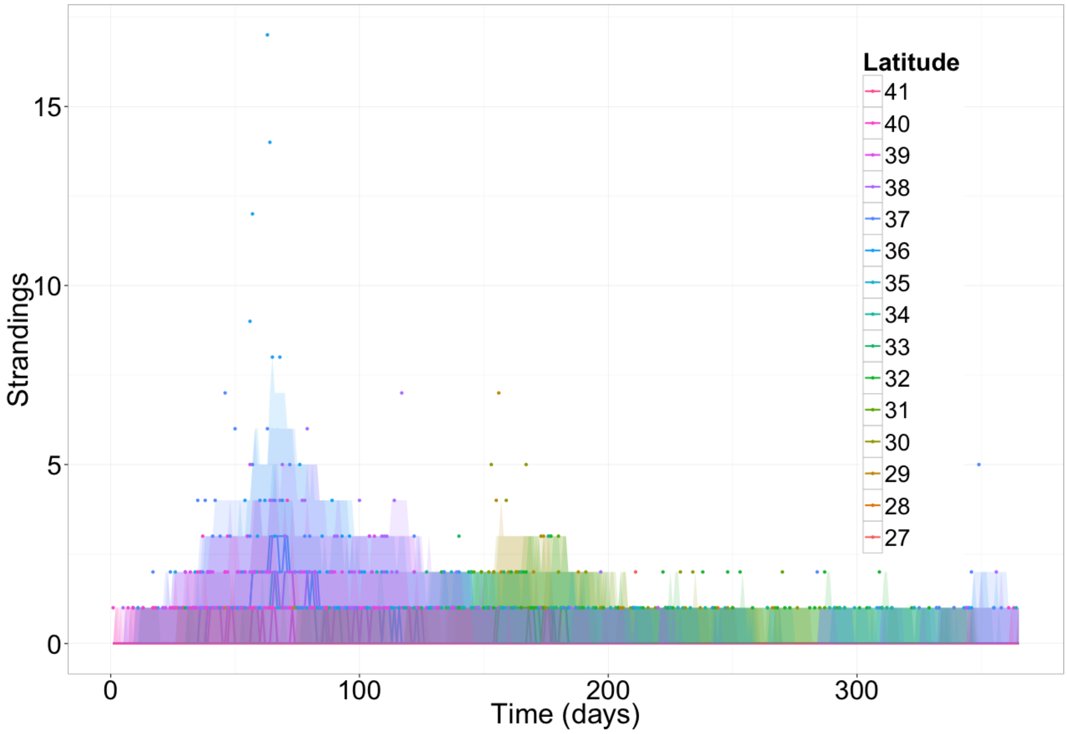

Figure S7. Simulated predictions of the frequency-dependent model. Cases were simulated across latitudes, l, and time, t, as $\mathrm{Pois}(\lambda_{l,t})$ using the hazard function, $\lambda_{l,t}$, calculated during parameter estimation. Lines represent median values from 2000 simulations in RStan, shaded regions represent the 2.5^{th}–97.5^{th} quantile range and points represent actual data. Latitude bands are distinguished by colour.

S6. Binomial chain model with susceptible depletion

A binomial chain version of the self-exciting process was developed to investigate the importance of susceptible depletion to the overall epidemic dynamics. In this model the hazard function is defined as

$$\lambda(t,l|H_t) = \beta \sum_{i,t_i<t} g(t-t_i) f(l-l_i)$$

for density-dependent transmission, and

$$\lambda(t,l|H_t) = \frac{\beta}{D_{t,l}} \sum_{i,t_i<t} g(t-t_i) f(l-l_i)$$

for frequency-dependent transmission. The number of susceptible individuals at time t and latitude l, $X_{t,l}$, is calculated by subtracting the cumulative number of strandings at l from the total population size, and then the number of new cases is drawn from a binomial distribution with $X_{t,l}$ trials and probability of success given by $p = 1 - \exp(-\lambda_{t,l})$. The effective reproduction number is $R = \beta X_{t,l}$ for density-dependent transmission and $R = \beta X_{t,l}/D_{t,l}$ for frequency-dependent transmission, and the functions f and g, and all other parameters, are as described in the main body of the text. Parameter estimates and information criterion values are given in Table S2, and model fits are shown in Figures S8 and S9.

Table S2. Model comparisons. Values indicate median parameter estimates and information criterion (2.5th–97.5th quantiles) from 2000 RStan simulations (not including warm-up sampling) of each model with susceptible depletion.

Model	Density-dependent	Frequency-dependent
R	min: 0.0071 (0.0067–0.0076)*<br>max: 2.87 (2.70–3.05)	min: 0.0877 (0.0871–0.0884)*<br>max: 0.99 (0.97–1.00)
σ	1.78 (1.80–1.85)	1.76 (1.75–1.78)
α	0.14 (0.11–0.18)	0.17 (0.14–0.21)
Number of parameters	3	3
Approximate AIC	6255.78	6014.03
WAIC	10982.09	12255.60

*Reproductive values are calculated using the inferred posterior distribution of β (results not shown). The minimum value refers to R at the lowest estimate of $X_{t,l}$ ($X_{t,l}/D_{t,l}$) in the density-dependent (frequency-dependent) case, for any one latitude degree and time point, and the maximum value refers to R at the largest estimate.

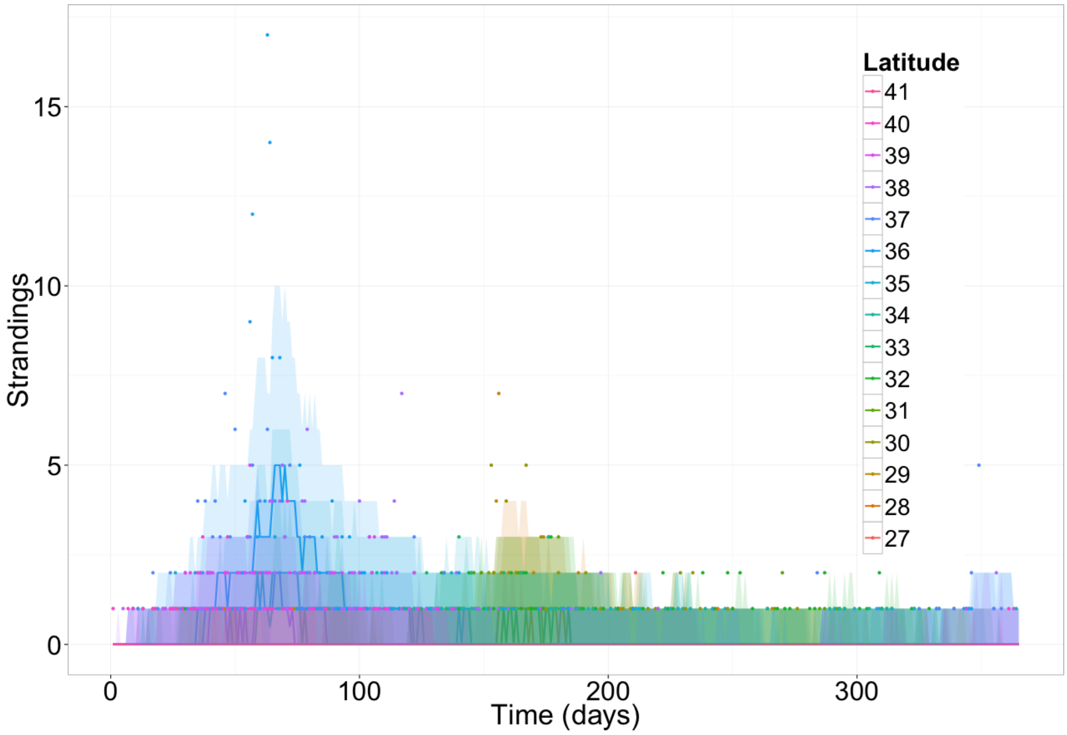

Figure S8. Simulated predictions of the density-dependent susceptible depletion model. Cases were simulated across latitudes, l, and time, t, as $\mathrm{Bi}(p = 1 - \mathrm{e}^{-\lambda_{t,l}}, X_{t,l})$ using the hazard function, $\lambda_{l,t}$, calculated during parameter estimation. Lines represent median values from 2000 simulations in RStan, shaded regions represent the 2.5th–97.5th quantile range and points represent actual data. Latitude bands are distinguished by colour.

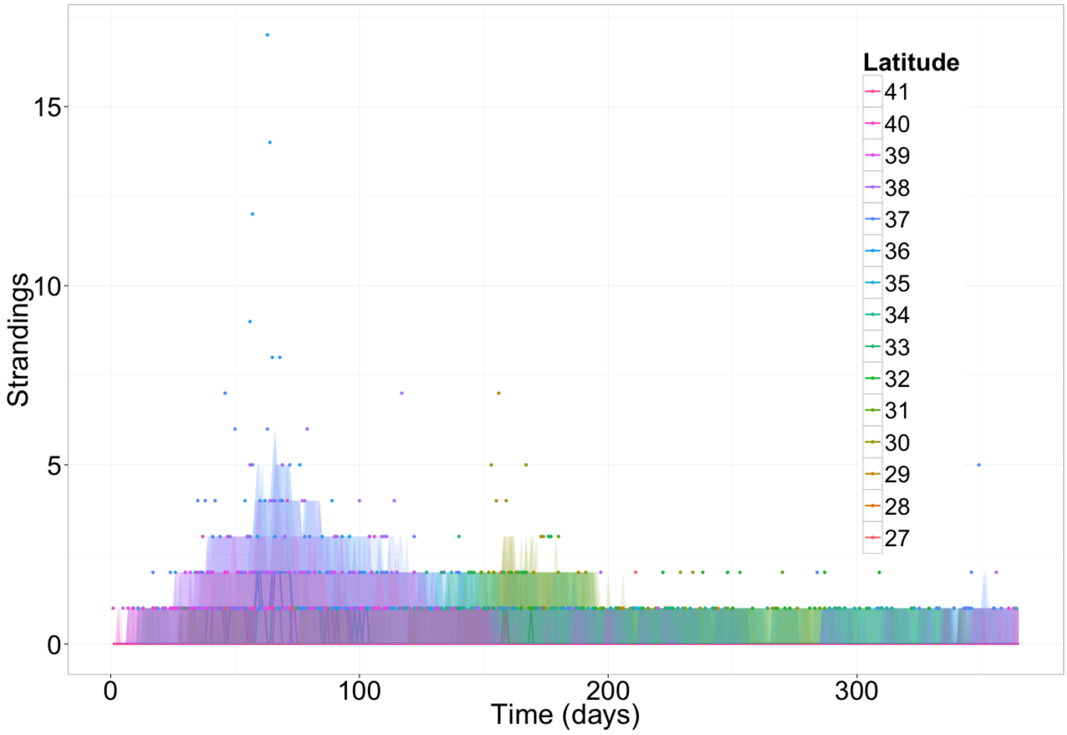

Figure S9. Simulated predictions of the frequency-dependent susceptible depletion model. Cases were simulated across latitudes, l, and time, t, as $\mathrm{Bi}(p = 1 - \mathrm{e}^{-\lambda_{t,l}}, X_{t,l})$ using the hazard function, $\lambda_{l,t}$, calculated during parameter estimation. Lines represent median values from 2000 simulations in RStan, shaded regions represent the 2.5^{th}–97.5^{th} quantile range and points represent actual data. Latitude bands are distinguished by colour.

S7. Transmission networks

S7.1. **Generating the networks.** As discussed in Section 2.4, transmission networks were reconstructed using the distribution of parameter estimates from the best-fitting model. Stranded individuals were connected with the source of their stranding (i.e. the previously stranded individual that most likely caused their infection) according to the following steps:

(1) One sample was taken from the joint posterior parameter distribution i.e. a value was drawn for each estimated parameter (the baseline and additional transmission rates, and the temporal and spatial decay rates). This sample was then used to reconstruct the hazard function (according to Equation 1), and the current contribution of each infected individual to the hazard, across all space and time points.

(2) For each individual i (that stranded at latitude l_i and time t_i):

i) The current contributions at l_i and t_i of each *previously stranded individual* (i.e. each potential source) were determined from the previous step.

ii) These contributions were divided by the total hazard function at l_i and t_i to determine the relative contribution of each potential source. A vector, C, representing the cumulative sum of these contributions was then created.

iii) Finally, the source was chosen using random number generation: a random number between 0 and 1, r, was drawn from a uniform distribution and the individual corresponding to the first element in C greater than r was identified as the source of infection.

(3) Step 2 was repeated for each stranded individual to generate the entire network.

A different transmission network was generated for each random sample of the estimated parameter distributions; 100 samples were taken in total. These transmission networks then served as a tool for visualizing and interpreting the results of the model inference.

S7.2. **Visualization.**

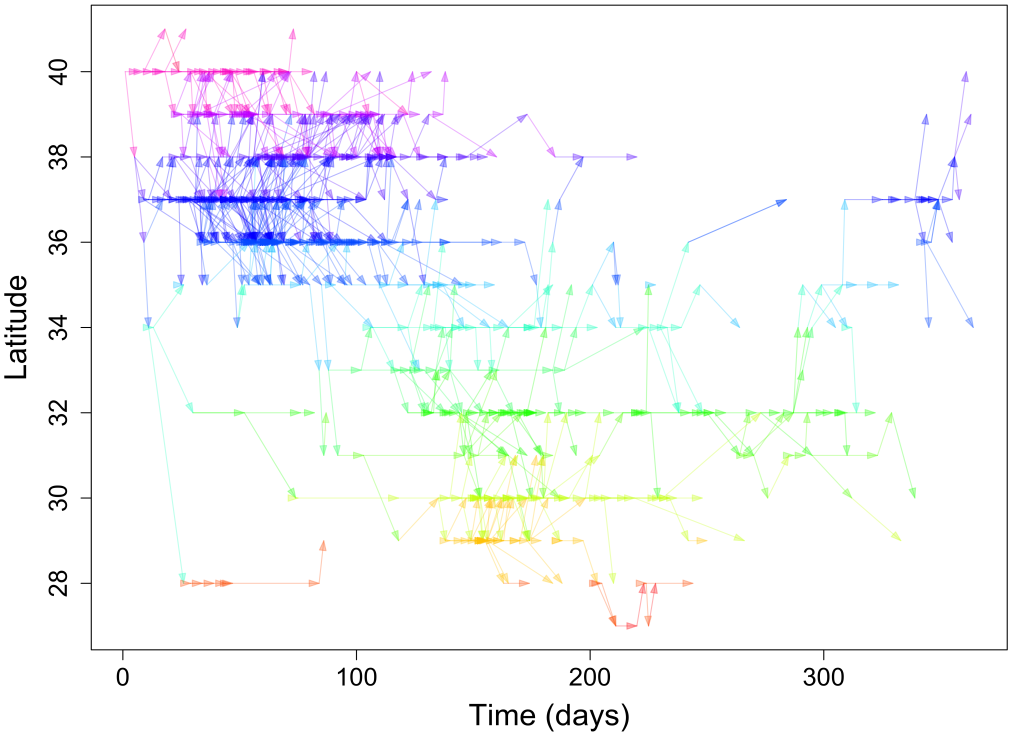

Figure S10. Example of one of the directed transmission networks. Each arrow represents the direction from the source of infection to the resulting stranding. Colours distinguish the different latitudes that generated each transmission event.

References

[1] D. J. Daley and D. Vere-Jones. *An introduction to the theory of point processess. Volume I: Elementary Theory and Methods, Second Edition.* New York: Springer, 2003.

[2] G. O. Mohler, M. B. Short, P. J. Brantingham, F. P. Schoenberg, and G. E. Tita. "Self-exciting point process modeling of crime." In: *Journal of the American Statistical Association* 106.493 (2011).

[3] H. Akaike. "A new look at the statistical model identification." In: *IEEE Transactions on Automatic Control* 19.6 (1974), pp. 716–723.

[4] A. Gelman, J. Hwang, and A. Vehtari. "Understanding predictive information criteria for Bayesian models." In: *Statistics and Computing* 24 (2014), pp. 997–1016.

[5] S. Watanabe. "Asymptotic equivalence of Bayes cross validation and widely applicable information criterion in singular learning theory." In: *The Journal of Machine Learning Research* 11 (2010), pp. 3571–3594.
